# Nutrition Concerns of Insufficient and Excessive Intake of Dietary Minerals in Lactating Women: A Cross-Sectional Survey in Three Cities of China

**DOI:** 10.1371/journal.pone.0146483

**Published:** 2016-01-05

**Authors:** Ai Zhao, Yong Xue, Yumei Zhang, Wenjun Li, Kai Yu, Peiyu Wang

**Affiliations:** 1 Department of Social Medicine and Health Education, School of Public Health, Peking University Health Science Center, Beijing, China; 2 Department of Nutrition & Food Hygiene, School of Public Health, Peking University Health Science Center, Beijing, China; 3 Nestlé Nutrition Institute, Beijing, China; 4 Nestlé Research Center Beijing, Beijing, China; School of Public Health, Zhejiang University, CHINA

## Abstract

**Objective:**

Objectives of this study were 1) to investigate the mineral intake by Chinese lactating women, 2) to explore the dietary source of minerals, and 3) the ratios between different dietary minerals.

**Methods:**

A total of 468 lactating women in 5–240 days post-partum participated in this study. Food intakes by participants were measured using one time of 24-hour dietary recall, and minerals from food were calculated based on the Chinese Food Composition Table, second edition.

**Results:**

In post-partum, women had inadequate food intake. 81.0% of women’s daily intake of dairy products was lower than 300g, and 97.1% of women’s daily intake of salt over 6g. For mineral intake, there were 81.8%, 59.0%, 47.6%, 45.7% and 66.8% of women’s calcium, magnesium, iron, zinc and selenium intake lower than the estimated average requirement, respectively, and 91.7% of women’s excessive intake of sodium. The calcium/phosphorus and sodium/potassium ratios were 0.41±0.26/1 and 3.13±2.89/1, respectively. Considering the dietary sources of minerals, 27.3%, 25.3% and 30.1% of iron, zinc and calcium were from animal-based food, respectively, and 60.3%, 66.1% and 58.0% of iron, zinc and calcium were from plant-based food, respectively. The phosphorus-protein ratio was 0.014±0.003/1. Lactation stage was associated with nutrient intake. Women within 30 days post-partum and the ones who live in Guangzhou had a significantly lower intake of certain minerals, while women with a high education experience had a high intake of calcium, potassium, iron and zinc. Productive age, whether obese or not, and delivery ways were not associated with mineral intakes (*P* all >0.05).

**Conclusion:**

Chinese women in three studied cities had an inappropriate food intake and resulted in both insufficient and excessive intakes of certain minerals.

## Introduction

Minerals are essential micronutrients for growth, development, and maintenance of healthy tissues. However, it has been reported that both in developed and developing countries there are a large part of lactating women did not meet the recommended mineral intake[1]. The long term insufficient intake of minerals may lead to bone demineralization, weaken the hemopoietic system and even life-threatening consequences [2]. In addition, some studies revealed that nursing mothers with mineral deficiency were more at risk to have low minerals’ contents in their breast-milk, which might affect early development of infants[3].

In China, a prevalent cultural practice in post-partum which includes a series of prescribed behaviors is adopted for the consideration of convalescence. The practice defined lots of “should do” activities, such as eating certain foods (the diet contains lots of poultry meat, eggs, and red sugar) and a number of “should not do” behaviors, such as bathing, lifting heavy objects and doing outdoor activities[4]. Although several studies have reported some foods in this practice are good for certain aspects of women’s recovery[5], one study in rural China suggested such a dietary pattern might lead to an insufficient intake of calcium during lactation[6]. For other minerals, whether the Chinese food habits during lactation can meet basic daily requirements or not are still unknown. In addition, food source and mineral to mineral interaction were directly related to the absorption of certain minerals[7]. Chinese traditional diet pattern was plant-based, which might influence the bioavailability of iron, calcium and zinc in the diet [7]. The ratios of certain minerals intake like calcium and phosphorus are also proved to can affect the bioavailability of calcium and even lead to health consequence [8–10]. This study was designed to measure the minerals’ intakes during lactation, to explore the dietary sources of minerals, and the ratios between different minerals.

## Materials and Methods

### Subjects

This study is part of the Maternal Infant Nutrition and Growth Study (MING Study) which is a cross-sectional survey carried out in 8 Chinese cities from August of 2011 to March of 2012. Studied lactating women were selected from 3 of the 8 cities (Beijing, Suzhou and Guangzhou) using a purposive sampling method (Beijing, Suzhou and Guangzhou were located in the north, middle and south of China respectively). One hospital and one community based maternal and child health care center were randomly selected in each city through a computer-generated hospitals list. Participants in each lactation stages were convenient recruited according to their visiting time until the number of participants satisfied the sample size. Finally 468 lactating women (Beijing, N = 168; Suzhou, N = 150 and Guangzhou, N = 150) during 5–240days post-partum (5–11days, N = 38; 12–30days, N = 90, 31–60days, N = 60, 61–120days, N = 100, 121–180days, N = 90 and 181–240-days, N = 90) were qualified and volunteered to participated in this study. The women were included with the following criteria: at the age of 18–45 years old, without metabolic diseases (diabetes, hypertension, hyperthyroidism and hypothyroidism), without using any hormone in recent three months and gave birth to a healthy full-term infant. The women who had post-partum depression were excluded. The women in 0–4 days post-partum were not involved in this study in order to minimize the influence on food intake by labor and hospital diet.

### Data collection

Data were collected from lactating women by trained interviewers using an interviewer-administered questionnaire with regard to demographic factors. Training of the interviewers, initial site survey, and preliminary questionnaire testing were completed prior to data collection.

One time 24-hour dietary recall was used to obtain the data of food intake. The standard bowls, plates and spoons were used for helping quantification of food consumption. Mineral compositions including calcium (Ca), phosphorus (P), potassium (K), sodium (Na), magnesium (Mg), iron (Fe), zinc (Zn), selenium (Se), copper (Cu) and manganese (Mn) and their dietary sources were analyzed based on the Chinese Food Composition Table second edition and the nutrient composition table on the food packaging[11]. The average intake amounts of the following foods in the previous 6 months were also estimated using semi-quantitative food frequency questionnaire: 1) grains, 2) vegetables, 3) fruits, 4) meat, 5) fish and shrimps, 6) eggs, 7) legumes, 8) nuts, 9) fats, and oils.

Weight and height were measured and used to calculate body mass index (BMI). BMI<18.5, 18.5–23.9, 24–27.9 and ≥28kg/m^2^ were considered as underweight, normal weight, over weight and obesity respectively according to Chinese BMI standard.

### Statistics

SPSS version 20.0 (SPSS Inc., Chicago, IL, USA) was used for analysis. Minerals intakes were compared with Chinese Dietary Reference Intakes (DRIs, 2013)[12]. The food average intakes were compared with the recommended daily food intake based on the Chinese balanced dietary pagoda[13]. Normality was tested for each data before analysis. Values were presented as Mean± SD, Median (25^th^,75^th^) or percentage. ANOVA and Chi-square analysis were used to compare demographic characters among women in different cities. Linear regression (with Enter method) was used to explore the potential factors(living cities, productive age, delivery ways, education experience and BMI) associated with minerals intake. Statistically significant difference in this study was set to P value <0.05.

### Ethics

This study was conducted according to the guidelines laid down in the Declaration of Helsinki and all procedures involving human participants were approved by the Medical Ethics Research Board of Peking University (NO.IRB00001052-11042). Written informed consent was obtained from all patients.

## Results

### Basic information

A total of 468 women in the 5–240days post-partum participated in the study. The demographic characteristics of lactating women among three cities were shown (Table 1). Lactating women in Beijing had a higher age, BMI and caesarean section rate, while had a higher education experience comparing with women in Suzhou and Guangzhou.

**Table 1:** Comparison of demographic characteristics of lactating women among three cities of China.

Variables		Total	Area			
			Beijing	Suzhou	Guangzhou	*P*
**Productive age**						
	Mean±SD y*	27.7±4.0	28.4±3.3	26.9±4.2	27.6±4.3	0.023
	< 25 y	126(56.8)	26(15.8)	55(37.2)	45(30.6)	0.001
	25–30 y	106(23.0)	95(57.6)	61(41.2)	65(44.2)	
	>30 y	93(20.2)	44(26.7)	32(21.6)	37(25.2)	
**BMI**						
	Mean±SD kg/m^2^*	23.2±3.1	24.1±3.2	23.3±3.0	22.2±2.9	<0.001
	Under weight	24(5.1)	8(4.8)	7(4.7)	9(6.0)	<0.001
	Normal weight	255(54.6)	74(44.3)	77(51.3)	104(69.3)	
	Over weight	152(32.5)	63(37.7)	58(38.7)	31(20.7)	
	obesity	36(7.7)	22(13.2)	8(5.3)	6(4.0)	
**Nationality**						0.094
	Han	448(95.7)	158(94.0)	148(98.7)	142(94.7)	
	Others	20(4.3)	10(6.0)	2(1.3)	8(5.3)	
**Delivery ways**						0.003
	Caesarean section	217(46.7)	91(54.2)	74(49.3)	53(35.3)	
	Vaginal delivery	248(53.3)	77(45.8)	76(51.7)	97(64.7)	
**Education experience**						<0.001
	Senior high or under	262(56.8)	26(15.8)	55(37.2)	45(30.6)	
	Bachelor degree	106(23.0)	95(57.6)	61(41.2)	65(44.2)	
	Master or above	93(20.2)	44(26.7)	32(21.6)	37(25.2)	

* Presented as Mean±SD and analyzed with ANOVA and other data were presented as N,% and analyzed with the method of Chi-square.

### Dietary intake

Compared with the recommendation of Chinese Balanced Dietary Pagoda, only a small proportion of women could consume appropriate amount of each group of food, especially for dairy products and salt(Table 2).

**Table 2:** The daily intake of different food groups by lactating women in three cities of China.

Food categories	N	Intakeamount(g) Median(25^th^,7^5th^)	Low intake (%) ^a^	Appropriate intake (%)^a^	High intake (%)^a^
Grains	449	320.0(241.5,431.4)	61.2	15.6	23.2
Vegetables	448	390.0(200.0,500.0)	44.6	34.8	20.5
Fruits	440	200.0(100.0,400.0)	43.2	35.5	21.4
Meat, fish &eggs	449	188.6(118.0,290.0)	53.7	22.9	23.4
Dairy products	342	200.0(71.4,250.0)	81.0	15.8	3.2
legumes & nuts	360	10.0(4.0,21.4)	83.9	8.9	7.2
Oils	446	28.0(21.0,35.0)	37.7	34.8	27.6
Salt	444	19.0(6.0,10.0)	2.9		97.1

a In comparison of food consumptions with Chinese Balanced Dietary Pagoda for lactating women, low intake, appropriate intake, and high intake for each food group are defined as:<350g, 350~450g, and >450g for grains; <300g, 300~500g, and >500g for vegetables; <200g, 200~400g, and >400g for fruits; <200g, 200~300g, and >300g for meat, fish and eggs; <300g, 300~500g, and >500g for dairy products; <40g, 40~60g, and >60g for legumes and nuts; <25g, 25~30g, and >30g for oils. For salt, ≤6g defined as appropriate intake, while >6g defined as high intake.

### Nutrients intake

Compared to recommendation in DRIs, insufficient intake and excessive intake of nutrients were both shown in the sampled subjects (Table 3). More than 50% of women insufficient intake of Ca, Mg and Se, while over 90% of women excessive intake of Na. The calcium-phosphorus (Ca/P) ratio in this study was 0.41±0.26/1 and the sodium-potassium (Na/K) ratio was 3.13±2.89/1.

**Table 3:** Dietary minerals intake by lactating women in three cities of China and comparison with Chinese Dietary Reference Intakes (estimated average requirement, EAR; recommended nutrient intake, RNI; adequate intake, AI, tolerable upper intake level, UL and proposed intakes for preventing non-communicable chronic disease, PI-NCD; N = 468).

Minerals	Mean	SD	25th	Media (50th)	75th	<EAR^a^%	<RNI/AI^b^%	>UL/PI-NCD^c^%
Calcium (mg)	512.6	416.6	246.6	391.5	661.7	81.8	89.5	1.1
Phosphorus (mg)	1076.9	541.0	682.2	964.4	1363.5	16.0	29.1	0.2
Potassium (mg)	1881.7	1070.4	1106.2	1682.2	2374.1	-	75.9	7.3
Sodium (mg)	4462.8	3275.0	3178.2	3854.7	4972.8	-	6.4	91.7
Magnesium (mg)	290.7	185.7	169.7	247.1	366.1	59.0	68.6	-
Iron (mg)	22.1	14.4	13.4	18.9	26.1	47.6	69.0	7.1
Zinc (mg)	11.6	6.1	7.2	10.5	14.0	45.7	61.9	0.4
Selenium (μg)	61.2	49.1	31.3	48.1	75.3	66.8	76.5	0.0
Copper (mg)	1.95	1.46	1.08	1.50	2.42	25.9	42.4	1.1
Manganese(mg)	5.19	5.83	2.99	4.10	6.04	-	60.2	4.3

a The EAR of calcium, phosphorus, magnesium, iron, zinc, selenium and copper were 810mg/d, 600mg/d, 280mg/d, 18mg/d, 9.9mg/d, 65μg/d and 1.1mg/d respectively.

b The RNI of calcium, phosphorus, magnesium, iron, zinc, selenium and copper were 1000mg/d, 720 mg/d, 330 mg/d, 24 mg/d, 12 mg/d, 78 μg/d and 1.4 mg/d respectively. The AI of manganese, sodium and potassium were 4.8mg/d, 1500mg/d and 2400mg/d respectively.

c The UL of calcium, phosphorus, potassium, iron, zinc, selenium, copper and manganese were 2000 mg/d, 3500 mg/d, 3600 mg/d, 42 mg/d, 40 mg/d, 400 μg/d, 8.0 mg/d and 11 mg/d, respectively. The PI-NCD of sodium was 2000mg/d.

### Source of minerals

**Fig 1:**
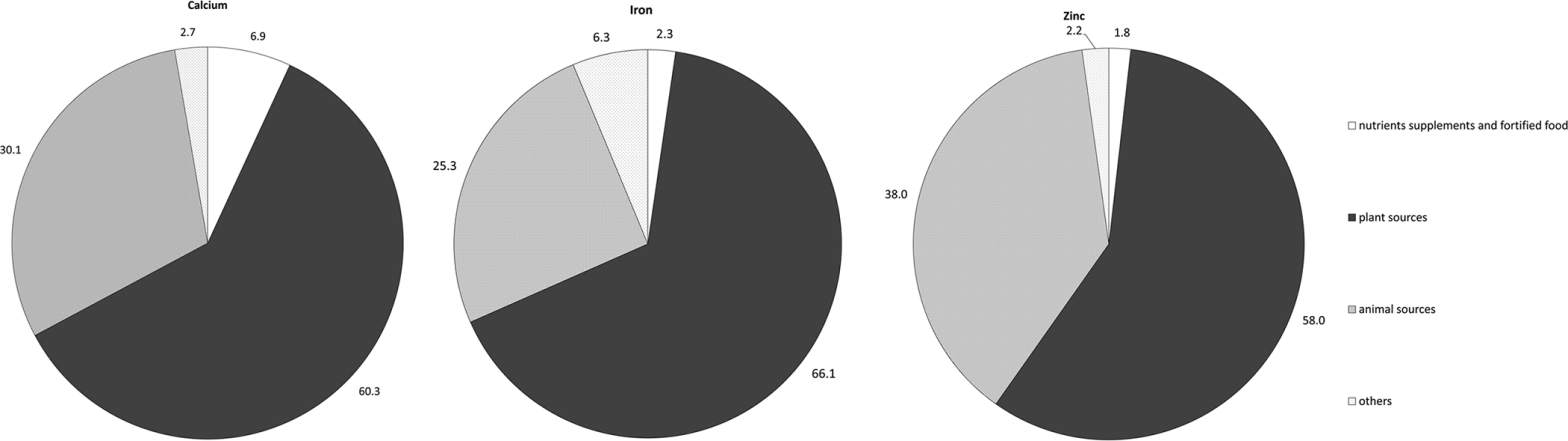
The dietary sources of calcium, iron and zinc in Chinese Lactating women (N = 468, Others included condiments and drinks). Calcium The percentage of each source of calcium intake; Iron The percentage of each source of iron intake; Zinc The percentage of each source of zinc intake.

For the dietary source of minerals, the study indicated that most of Ca intake was plant food, only a small proportion of Ca was from nutrient supplements and fortified food and 25.3% and 38.0% of dietary Fe and Zn respectively were obtained from animal origin (Fig 1). Additionally, a great part of P was in its inorganic form, and the phosphorus-protein (P/Pro) ratio was 0.014±0.003/1 (the average daily protein intake by this population was 78.9 ± 46.2g).

### Factors effect on nutrients intake

**Fig 2:**
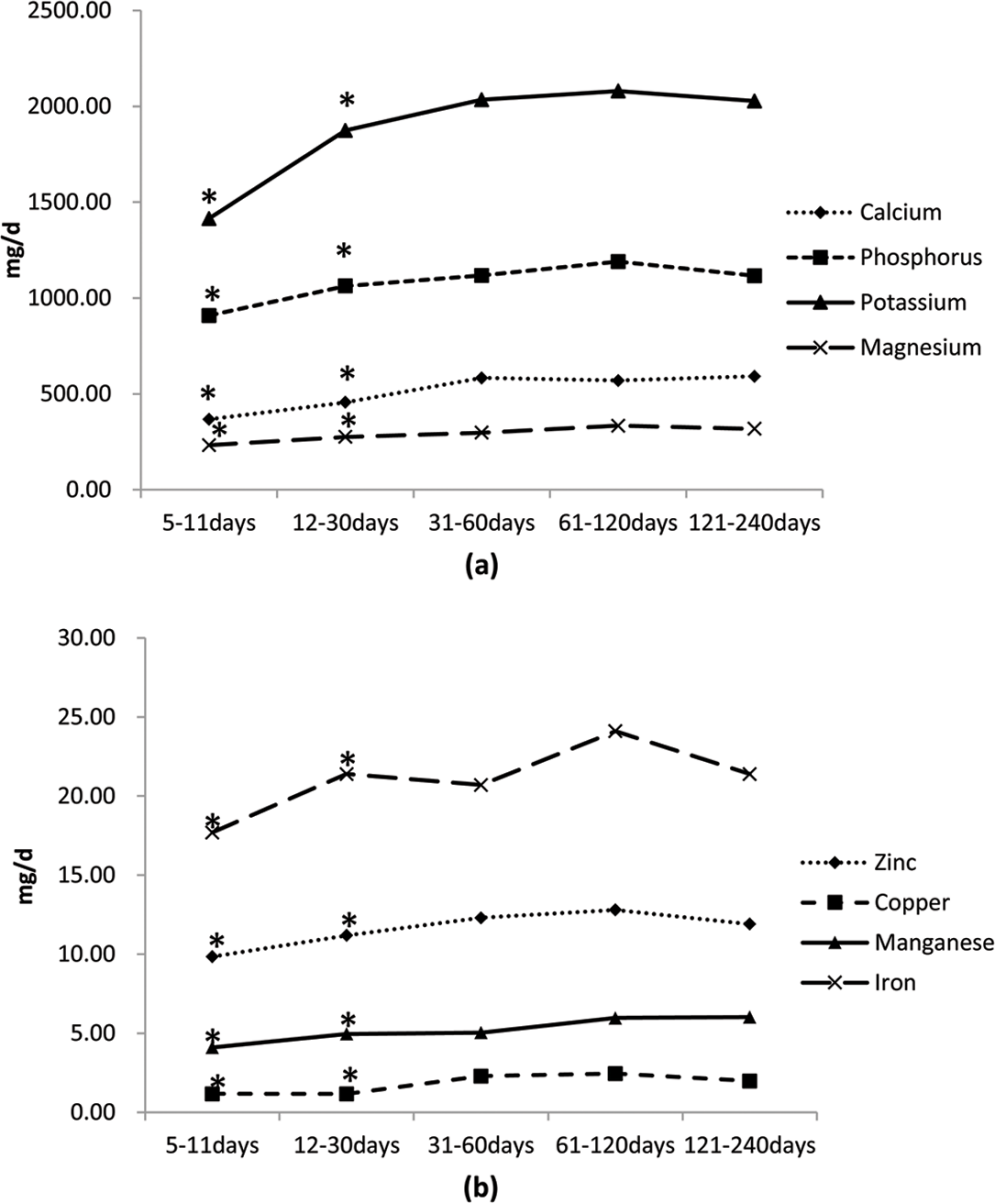
The changes of minerals intake of lactating women through different lactation stages (N = 468, * significant differences compared with other lactation stages). (a) Lactating women in 5–11days and 12–30days postpartum had a significant lower intake of calcium, phosphorus, potassium and magnesium comparing women in the 31–60day, 61–120days and 121–240days post-partum.(b)Lactating women in 5–11days and 12–30days postpartum had a significant lower intake of zinc, copper, manganese and iron comparing women in the 31–60day, 61–120days and 121–240days post-partum.

Factors might affect minerals intake were analyzed by linear regression model. Variables of areas defined as 1 = Beijing, 2 = Guangzhou, and 3 = Suzhou; lactation stages defined as 1 = 5–11days, 2 = 12–30days, 3 = 31–60days,4 = 61–120days, and 5 = 121–240days; age defined as 1 = under 25y, 2 = 25–29.9y, and 3 = 30y or above; BMI defined as 1 = underweight, 2 = normal weight, 3 = overweight and 4 = obesity; delivery ways defined as 1 = vaginal delivery and 2 = ceasarean section and education experience defined as 1 = senior high school or under, 2 = bachelor degree, and 3 = master degree or above. Area differences were showed in Ca, K, Mg, Fe and Cu; while Ca, P, K, Mg, Zn, Cu and Mn were showed had time differences. Education experience were linear associated with minerals intakes of Ca, P, K, Fe and Zn. Productive age, obesity or not and delivery ways were not associated with minerals intakes (Table 4). According to ANOVA analysis and LSD post hoc analysis, Lactating women within 5–30days post-partum had significantly lower minerals intake and there were no significant differences of minerals intake among other lactation stages (Fig 2). Considering living cities, lactating women in Guangzhou had a significant lower intake of Ca, K, Mg, Fe and Cu (Table 5). Lactating women who had a master or above degree education experience had a higher intake of Ca, K, Fe and Zn (*P* all<0.05).

**Table 4:** Linear regression of factors associated with minerals intake.

Minerals	Area		Lactation Stage		Productive age		BMI		Delivery ways		Education experience	
	t	*P*	t	*P*	t	*P*	t	*P*	t	*P*	t	*P*
Calcium	-3.299	0.001	4.881	<0.001	0.903	0.182	-0.434	0.664	0.218	0.827	3.092	0.002
Phosphorus	-1.122	0.262	3.990	<0.001	0.074	0.818	0.613	0.541	-1.195	0.233	2.072	0.039
Potassium	-2.150	0.032	5.265	<0.001	0.917	0.209	-0.404	0.686	-0.602	0.548	2.812	0.005
Sodium	-0.958	0.338	1.933	0.054	-0.862	0.534	0.195	0.846	-1.110	0.268	1.314	0.190
Magnesium	-2.825	0.005	4.626	<0.001	0.704	0.367	0.347	0.729	-0.298	0.776	1.447	0.149
Iron	-2.143	0.033	2.263	0.024	-0.128	0.797	0.624	0.533	0.752	0.452	2.671	0.008
Zinc	0.585	0.559	3.593	0.001	0.279	0.538	0.076	0.939	0.241	0.809	2.429	0.018
Selenium	-1.086	0.278	-0.075	0.940	0.240	0.995	0.682	0.496	-0.456	0.649	0.144	0.885
Copper	-3.352	0.001	3.854	<0.001	1.233	0.147	-1.018	0.309	1.271	0.204	1.499	0.135
Manganese	-1.347	0.179	2.791	0.005	0.565	0.561	-0.986	0.325	0.523	0.601	-0.038	0.970

**Table 5:** Differences of minerals intake of lactating women among different cities in China.

	Minerals intake		
	Beijing N = 168	Suzhou N = 150	Guangzhou N = 150
Calcium (mg)	553.9±426.4	592.5±469.9	386.4±308.1*
Phosphorus (mg)	1050.4 ±496.6	1234.8±580.2	948.7±511.7
Potassium (mg)	1868.7±959.5	2203.5±1148.2	1574.3±1020.3*
Sodium (mg)	4922.9±4351.9	3872.3±2737.9	4538.0±2073.0
Magnesium (mg)	299.4±214.7	337.0±162.4	234.6±156.9*
Iron (mg)	23.3±16.0	24.3±15.2	18.7±10.8*
Zinc (mg)	10.8±5.1	13.3±6.8	10.9±9.2
Selenium (μg)	59.6±39.7	74.1±57.4	50.2±46.7
Copper (mg)	2.09±1.56	2.19±1.54	1.56±1.16*
Manganese(mg)	4.93±6.42	6.37±7.19	4.32±2.53

*Post hoc LSD test shows lower intake of minerals compared with other cities.

## Discussion

The requirements for minerals during lactation period are quantitatively greater than those during pregnancy and recommendation level for adults, therefore lactation poses a significant threat to maternal mineral homeostasis[14]. Previous studies reported a high prevalence of mineral deficiencies in lactating women, particularly in women with chronically low dietary minerals intakes[15]. The current study has supported and extended previous findings by demonstrating that Chinese women during lactation had an inappropriate food intake, and although the intake of some minerals has been improved, women still faced the nutrition problems of both insufficient and excessive intake of certain minerals.

One of the most concerned nutrition problems in Chinese lactating women was the low intake of Ca. The average daily Ca intake by the studied population was only 512.6mg, which was slight higher than the result performed in the rural areas of China (493mg/d)[6]. However it was far lower than the studies in other countries [16, 17]. Diet with low intake of dairy products accounts for the inadequate intake of Ca. In this study, over 80% of women could not intake sufficient dairy products. When dietary Ca intake was insufficient, Ca supplement was encouraged during lactation[18]. However, in this study only 2.7% of Ca was from nutrient supplements or fortified foods. Actually, the requirement for Ca during lactation is greater than any other adult life periods. Metabolic adjustments may occur in lactation to ensure that Ca is conserved and channeled to the breast for milk production. We estimated that the daily excretion of Ca from breast-milk was around 200 mg (MING study reported the Ca concentration in breast milk was around 250mg/kg[19], and infants of 0–8month old consumed around 0.8kg breast milk per day[20]), which was a substantial proportion of dietary Ca intakes in this study. In addition, we also found a high intake of P with a high absorptivity (Most of P were in inorganic form). The high P intake may not only lead to hyperphosphatemia but also considerably affect the Ca absorption and result in osteodystrophy[8]. The previous studies showed a high dietary Ca/P ratio was positively related to bone mass density[9, 10].The intake of Ca/P ratio in this study was only 0.41±0.26/1. The low intake of Ca/P ratio might aggravate the Ca deficiency in lactating women. In conclusion, an insufficient intake of dietary Ca without inclusion of Ca supplement, accompanied with a low Ca/P ratio, lactating women in studied cities might face a risk of Ca deficiency.

Another nutrition concern in this studied population was the high Na intake with an insufficient intake of K. Na and K are important electrolytes for human body to maintain blood pressure, adjust extracellular fluid capacity and constitute extracellular fluid osmotic pressure. All of the hypernatremia, hyponatremia, hyperkalemia and hypokalemia can seriously affect human health[21]. This study indicated that the dietary Na/K ratio was 3.13±2.89/1. The similar ratio was reported in a study performed in Zhejiang province, China[22]. And in some Asian counties, like Korea, which also reported a high Na intake (around 4.7g/d), while K intake was higher than our finding (K intake was around 2.9g/d and the Na/K ratio was 1.7:1)[23]. The major dietary Na source in China was salt. Huang et al. reported that in Zhejiang province, China, there was 82% of Na from salt [22]. This study identified 97.1% of women daily intake salt over 6 g. The high ratio of Na and K in this study might imply a series of health consequences such as obesity, renal stones, osteoporosis and stomach cancer[24].

The current study found the intakes of Fe and Zn in Chinese lactating women were improved. The intake amount of Fe and Zn were higher than the reported level in another study[6]. Considering the nutrient source, 25.3% of Fe was obtained from animal origin, which was also higher than the previous report (13.78% in Chinese adults)[25]. However, it’s worth noting that studied Chinese lactating mothers still face the threat of insufficient intake of Fe and Zn, because of nearly half of the studied subjects intakes of Fe and Zn lower than EAR.

This study also indicated that women within 5–30days post-partum had a significant lower nutrients intake. This result might be due to the special Chinese ritual within 30days post-partum. Although the traditional Chinese diet in post-partum was believed can restore maternal post-partum health and prevent future disease[26]. And some of the certain food intake in post-partum like chicken soup was proved can improve nursing performance[5]. However this study revealed that traditional Chinese diet in post-partum might also lead threat to nutrition deficiencies. The diversity of food intake was also found among cities and women with different education experience. These results suggested nutritional education should be conducted to lactating women and should be in accordance with local food habit.

### Limitation

Because of the cross-sectional design, inherent limitations of this study were unavoidable. And this survey was carried out through summer to winter. The seasonality in food consumption might exist, especially in northern city such as Beijing. However, we inferred this variation was very limited, because of all the participants were from urban areas and had good food diversity.

For the methodology, one time 24 hours dietary recall was used in this study to measure the food intake and estimate the minerals intake levels on a daily basis. Although a three-days or seven-days dietary recall were better to measure the daily variance of food intake, one time of dietary recall used in this study can also present the daily minerals intake by lactating women, because of the food intake by Chinese lactating women were usually homogeneous by the food restriction of the traditional culture. In addition, although standard bowls, plates and spoons were used for helping quantification of food consumption, recall bias might still exist.

In this study we did not obtain blood or hair samples from participants, the nutrient deficiency rate in lactating women were unknown. In addition, whether the low intake of calcium will result in the bone calcium loss or not could not been known. Further studies will be still needed to explore the short term and long term effects of mineral intake on health of Chinese lactating women.

### Conclusion

Based on the results in this study, multiple nutrition concerns were found in Chinese lactating women and might affect the maternal health and their offspring, especially for women in 5–30 post-partum. Several suggestions could be considered to improve maternal health: 1) Encourage women to intake adequate amount of dairy products on a daily basis (≥300g, in most cases, be met by increasing milk consumption by 3–4 cup/day); 2) Add adequate amount of Ca supplements if diet is lack of Ca. 3) Reduce the salt used in cooking. 4) Encourage to eat more food rich in iron and zinc. An appropriate combination of interventions may be an ideal way to reach an overall nutrition improvement for Chinese lactating women.

## Supporting Information

S1 Database The database used in this study. (SAV)

S1 Table Minerals intake among women with different education experience. Minerals intake among women with different education were compared with the method of ANOVA analysis and Post hoc LSD tests. *Indicates the significant minerals intake differences of women with master or above degree comparing with women with other education experience.
(PDF)
