# Supplementary material for: Nutrition Concerns of Insufficient and Excessive Intake of Dietary Minerals in Lactating Women: A Cross-Sectional Survey in Three Cities of China
Source: PLoS One. 2016 Jan 5;11(1):e0146483. doi: 10.1371/journal.pone.0146483 (PMC4701389; doi:10.1371/journal.pone.0146483)
Supplement: S1 Table — Minerals intake among women with different education were compared with the method of ANOVA analysis and Post hoc LSD tests. *Indicates the significant minerals intake differences of women with master or above degree comparing with women with other education experience. (PDF) [file pone.0146483.s002.pdf]

S1 Table. Differences of minerals intake among women with different education experience

	Minerals intake		
	Beijing N=262	Suzhou N=102	Guangzhou N=93
Calcium (mg)	462.0±415.7	527.5±414.5	640.1±407.9*
Phosphorus (mg)	1046.9±536.0	1071.5±548.3	1169.2±551.5
Potassium (mg)	1768.4±960.0	1975.0±1242.9	2112.7±1142.5*
Sodium (mg)	4321.9±2628.8	4542.1±2806.9	4789.6±5007.6
Magnesium (mg)	279.4±195.6	297.0±182.3	316.2±173.0*
Iron (mg)	20.9±11.6	21.4±14.3	26.6±20.4*
Zinc (mg)	11.3±5.9	11.5±5.9	12.7±7.2*
Selenium (μg)	60.8±54.0	60.8±42.4	63.2±41.4
Copper (mg)	1.82±1.29	2.14±1.77	2.12±1.47
Manganese(mg)	5.10±5.32	5.64±8.49	4.96±3.02

*Post hoc LSD test shows lower intakes of minerals compared with women lower education experience.
